# Bis{4′-[(2,3,5,6,8,9,11,12-octa­hydro-1,4,7,10,13-benzopenta­oxacyclo­penta­decin-15-yl)meth­oxy]-2,2′:6′,2′′-terpyridine}cadmium(II) bis­(hexa­fluorido­phosphate) trihydrate: a powder study

**DOI:** 10.1107/S1600536809040926

**Published:** 2009-10-10

**Authors:** Nadezhda M. Kurochkina, Andrey S. Kuzovlev, Tamara P. Puryaeva, Yurii A. Velikodny, Vladimir V. Chernyshev

**Affiliations:** a A.N. Frumkin Institute of Physical Chemistry and Electrochemistry, Leninsky prospect 31, 119991 Moscow GSP-1, Russian Federation; b Department of Chemistry, Moscow State University, 119991 Moscow, Russian Federation

## Abstract

The asymmetric unit of the title compound, [Cd(C_30_H_31_N_3_O_6_)_2_](PF_6_)_2_·3H_2_O, contains one half-cation with the Cd^II^ center situated on a twofold rotational axis, one hexa­fluoridophosphate anion and two uncoordinated water mol­ecules, one of which is also situated on a twofold rotational axis. The cations are associated into columns along the *a* axis through π–π inter­actions between the pyridine and benzene rings, with a centroid–centroid distance of 3.72 (5) Å. Inter­molecular O—H⋯O, C—H⋯O and C—H⋯F hydrogen bonds consolidate the crystal packing.

## Related literature

For the crystal structures of related complexes with the 4′-(4′′′-benzo-15-crown-5)-meth­yloxy-2,2′:6′,2′′-terpyridine ligand, see: Tsivadze *et al.* (2008 ▶); Logacheva *et al.* (2009 ▶). For details of the indexing algorithm, see: Visser (1969 ▶). 
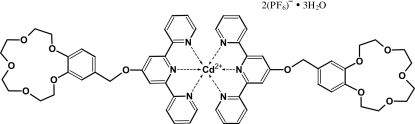


## Experimental

### 

#### Crystal data

[Cd(C_30_H_31_N_3_O_6_)_2_](PF_6_)_2_·3H_2_O
*M* *_r_* = 1515.54
Orthorhombic, 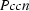

*a* = 12.720 (3) Å
*b* = 21.101 (3) Å
*c* = 24.795 (5) Å
*V* = 6655 (2) Å^3^
*Z* = 4
Cu *K*α_1_ radiation
μ = 3.98 mm^−1^
*T* = 295 K
Specimen shape: flat sheet
15 × 1 × 1 mm
Specimen prepared at 101 kPa
Specimen prepared at 295 K
Particle morphology: no specific habit, colourless

#### Data collection

Guinier camera G670 diffractometer
Specimen mounting: thin layer in the specimen holder of the camera
Specimen mounted in transmission mode
Scan method: continuous
2θ_min_ = 4.0, 2θ_max_ = 80.0°
Increment in 2θ = 0.01°

#### Refinement

*R*_p_ = 0.020
*R*_wp_ = 0.024
*R*_exp_ = 0.015
*R*_B_ = 0.061
*S* = 1.67
Wavelength of incident radiation: 1.54059 Å
Profile function: split-type pseudo-Voigt (Toraya, 1986 ▶)
2031 reflections
184 parameters
197 restraints
H-atom parameters not refined
Preferred orientation correction: none

### 

Data collection: *Huber G640* (Huber, 2002 ▶); cell refinement: *MRIA* (Zlokazov & Chernyshev, 1992 ▶); data reduction: *Huber G640* (Huber, 2002 ▶); method used to solve structure: simulated annealing (Zhukov *et al.*, 2001 ▶); program(s) used to refine structure: *MRIA*; molecular graphics: *PLATON* (Spek, 2009 ▶); software used to prepare material for publication: *MRIA* and *SHELXL97* (Sheldrick, 2008 ▶).

## Supplementary Material

Crystal structure: contains datablocks I, global. DOI: 10.1107/S1600536809040926/er2073sup1.cif

Rietveld powder data: contains datablocks I. DOI: 10.1107/S1600536809040926/er2073Isup2.rtv

Additional supplementary materials:  crystallographic information; 3D view; checkCIF report

## Figures and Tables

**Table 1:** Hydrogen-bond geometry (Å, °)

*D*—H⋯*A*	*D*—H	H⋯*A*	*D*⋯*A*	*D*—H⋯*A*
O1*W*—H1*W*⋯O2*W*	0.85	2.08	2.91 (6)	165
C3—H3⋯F15^i^	0.93	2.44	3.21 (7)	140
C4—H4⋯O1*W*^ii^	0.93	2.23	3.17 (4)	178
C4—H4⋯O1*W*^iii^	0.93	2.23	3.17 (4)	178
C7—H7⋯O1*W*^ii^	0.93	2.28	3.20 (7)	175
C7—H7⋯O1*W*^iii^	0.93	2.28	3.20 (7)	175
C12—H12⋯F13^iv^	0.93	2.44	3.10 (7)	128
C15—H15⋯O4^iv^	0.93	2.51	3.23 (8)	135
C22—H22⋯O5^v^	0.93	2.60	3.31 (10)	133
C23—H23*B*⋯F15^vi^	0.97	2.36	3.23 (8)	149
C30—H30*B*⋯F11^ii^	0.97	2.46	3.23 (8)	137

Symmetry codes: (i) 
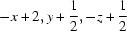
; (ii) 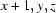; (iii) 
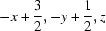
; (iv) 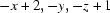; (v) 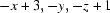; (vi) 
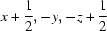
.

**Fig. 1.**
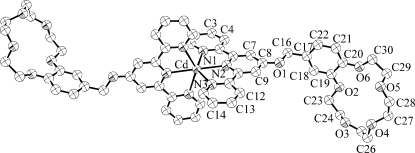
The molecular structure of the cation in (I) with the atomic numbering and 40% displacement spheres. Unlabelled atoms are related with the labeled ones by symmetry operation (3/2 - x, 1/2 - x, z). H atoms omitted for clarity.

**Fig. 2.**
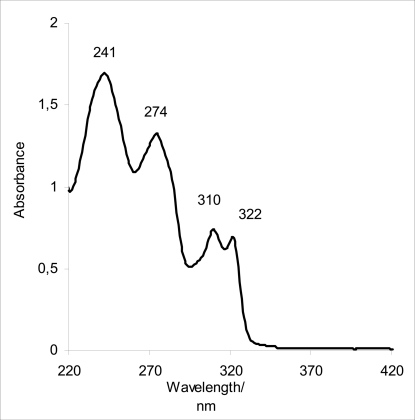
The UV-Vis spectrum of the [CdL2].2(PF6).3H2O in acetonitrile.

**Fig. 3.**
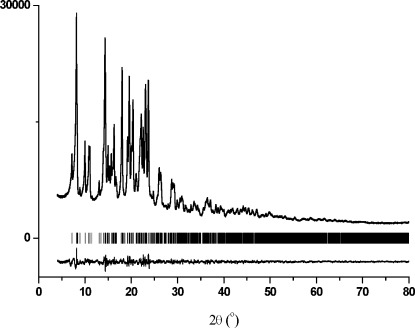
The Rietveld plot, showing the observed and difference profiles for (I). The reflection positions are shown above the difference profile.

## supplementary crystallographic information

### Comment

In continuation of our study of complexes with a hybrid ligand 4'-(4'''-benzo-15-crown-5)-methyloxy-2,2':6',2''-terpyridine (*L*) (Tsivadze *et al.*, 2008; Logacheva *et al.*, 2009) we present here the title compound (I), which is isostructural with the analogue Co and Zn complexes (Logacheva *et al.*, 2009).

In the cation of (I) (Fig. 1), the coordinating Cd—N bond lengths are 2.34 (3), 2.39 (6) and 2.42 (6) Å, respectively. The cations are associated into columns along axis *a* through π-π interactions between the pyridine and benzene rings with the centroid-to-centroid distance of 3.72 (5) Å. Intermolecular O—H···O, C—H···O and C—H···F hydrogen bonds (Table 1) consolidate the crystal packing.

The electronic absorption spectra in the visible and UV regions of acetonitrile solution of (I) (Fig. 2) were recorded on a Varian Cary-100 spectrophotometer in rectangular quartz cells with a path length of 10 mm.

### Experimental

Acetonitrile (Reagent ACS), methanol (for HPLC), ethanol (anhydrous), NH4PF6 (99%) were purchased from commercial supplier (Acros Organics). Cd(CH_3_COO)_2_.2H_2_O of high-purity grade was domestically produced. 4'-(4'''-Benzo-15-crown-5)-methyloxy-2,2':6',2''-terpyridine (*L*) was synthesized as described by Tsivadze *et al.* (2008).

[Cd*L*_2]_(PF_6_)_2_.3H_2_O: to a stirred solution of Cd(CH_3_COO)_2_.2H_2_O (95.7 mg, 0.1890 mmol) in methanol (15 ml) was added 40 ml of *L* (190.0 mg, 0.3759 mmol) in methanol. The mixture was stirred for 2 h and then NH_4_PF_6_ (1.23 g, 7.558 mmol) in 20 ml me thanol was added. Upon addition of ammonium hexafluoridophosphate a colourless solid was obtained that was then filtred, washed with methanol and diethyl ether, and dried. Subsequent recrystallization from mixture of EtOH: CH_3_CN (3: 1) gave (I) as colourless fine crystalline powder. Yield: 295.8 mg (56.4%). Anal. Calcd for C_60_H_68_F_12_CdN_6_O_15_P_2_: C, 47.55; H, 4.52; N, 5.55. Found: C, 47.18; H, 4.69; N, 5.26.

^1^H NMR spectra were recorded on a Bruker Avance-600 spectrometer operating at 600 MHz with internal deuterium lock at room temperature. The residual proton signal from DMSO-d_6_ (2.50 p.p.m.) was used as the internal reference for measuring ^1^H NMR chemical shifts. ^1^H NMR (600 MHz, DMSO-d_6_), δ, p.p.m.: 3.56–3.68 (m, 16H, c, c', d, d'), 3.79–3.86 (m, 8H, b, b'), 4.04–4.16 (m, 8H, a, a'), 5.49 (s, 4H, –CH_2_-benzyl), 7.04 (d J_β__-__γ_ = 7.88 Hz, 2H, γ), 7.16 (dd unres., 2H, β), 7.22 (d unres., 2H, α), 7.45–7.71 (m, 4H, 5,5''), 8.05–8.35 (m, 8H, 6,6'',4,4''), 8.51 (s br., 4H, 3',5'), 8.71–8.97 (m, 4H, 3,3'').

### Refinement

During the exposure, the specimen was spun in its plane to improve particle statistics. The orthorhombic unit-cell dimensions were determined with the indexing program ITO (Visser, 1969), *M*_20_=34, using the first 35 peak positions. The structure of (I) was solved by simulated annealing procedure (Zhukov *et al.*, 2001) and refined following the methodology described in details elsewhere (Logacheva *et al.*, 2009) by the subsequent bond-restrained Rietveld refinement with the program MRIA (Zlokazov & Chernyshev, 1992). Six *U*_iso_ parameters were refined - one for Cd, overall *U*_iso_ for non-H atoms from *L*, one for P, overall *U*_iso_ for six F atoms and two parameters for two water' O atoms. All H atoms were placed in geometrically calculated positions and not refined. The diffraction profiles and the differences between the measured and calculated profiles are shown in Fig. 3.

### Crystal data

**Table:**

[Cd(C_30_H_31_N_3_O_6_)_2_](PF_6_)_2_·3H_2_O	*F*(000) = 3104
*M**_r_* = 1515.54	*D*_x_ = 1.513 Mg m^−^^3^
Orthorhombic, *P**c**c**n*	Cu *K*α_1_ radiation, λ = 1.54059 Å
Hall symbol: -P 2ab 2ac	µ = 3.98 mm^−^^1^
*a* = 12.720 (3) Å	*T* = 295 K
*b* = 21.101 (3) Å	Particle morphology: no specific habit
*c* = 24.795 (5) Å	colourless
*V* = 6655 (2) Å^3^	flat_sheet, 15 × 1 mm
*Z* = 4	Specimen preparation: Prepared at 295 K and 101 kPa

### Data collection

**Table:**

Guinier camera G670 diffractometer	Data collection mode: transmission
Radiation source: line-focus sealed tube	Scan method: continuous
Curved Germanium (111)	2θ_min_ = 4.00°, 2θ_max_ = 80.00°, 2θ_step_ = 0.01°
Specimen mounting: thin layer in the specimen holder of the camera	

### Refinement

**Table:**

Refinement on *I*_net_	184 parameters
Least-squares matrix: full with fixed elements per cycle	197 restraints
*R*_p_ = 0.020	45 constraints
*R*_wp_ = 0.024	H-atom parameters not refined
*R*_exp_ = 0.015	Weighting scheme based on measured s.u.'s
*R*_Bragg_ = 0.061	(Δ/σ)_max_ = 0.004
χ^2^ = 2.785	Background function: Chebyshev polynomial up to the 5th order
7601 data points	Preferred orientation correction: none
Profile function: split-type pseudo-Voigt (Toraya, 1986)	

### Special details

**Table:**

Geometry. All e.s.d.'s (except the e.s.d. in the dihedral angle between two l.s. planes) are estimated using the full covariance matrix. The cell e.s.d.'s are taken into account individually in the estimation of e.s.d.'s in distances, angles and torsion angles; correlations between e.s.d.'s in cell parameters are only used when they are defined by crystal symmetry. An approximate (isotropic) treatment of cell e.s.d.'s is used for estimating e.s.d.'s involving l.s. planes.

### Fractional atomic coordinates and isotropic or equivalent isotropic displacement parameters (Å^2^)

**Table:**

	*x*	*y*	*z*	*U*_iso_*/*U*_eq_	
Cd	0.7500	0.2500	0.4194 (14)	0.058 (5)*	
O1	1.200 (2)	0.111 (2)	0.408 (3)	0.076 (6)*	
O2	1.459 (2)	−0.076 (2)	0.327 (3)	0.076 (6)*	
O3	1.369 (2)	−0.199 (3)	0.328 (3)	0.076 (6)*	
O4	1.460 (2)	−0.257 (3)	0.426 (3)	0.076 (6)*	
O5	1.6125 (19)	−0.161 (2)	0.470 (3)	0.076 (6)*	
O6	1.606 (2)	−0.056 (2)	0.395 (3)	0.076 (6)*	
N1	0.859 (2)	0.304 (2)	0.355 (3)	0.076 (6)*	
N2	0.917 (2)	0.204 (3)	0.416 (3)	0.076 (6)*	
N3	0.761 (2)	0.166 (2)	0.484 (3)	0.076 (6)*	
C1	0.826 (2)	0.354 (3)	0.325 (3)	0.076 (6)*	
H1	0.7590	0.3698	0.3309	0.091*	
C2	0.887 (2)	0.382 (3)	0.285 (3)	0.076 (6)*	
H2	0.8634	0.4173	0.2664	0.091*	
C3	0.986 (3)	0.356 (3)	0.274 (3)	0.076 (6)*	
H3	1.0274	0.3718	0.2464	0.091*	
C4	1.021 (2)	0.305 (3)	0.305 (3)	0.076 (6)*	
H4	1.0878	0.2879	0.2996	0.091*	
C5	0.955 (2)	0.278 (3)	0.344 (3)	0.076 (6)*	
C6	0.989 (2)	0.225 (3)	0.380 (3)	0.076 (6)*	
C7	1.087 (3)	0.195 (3)	0.373 (3)	0.076 (6)*	
H7	1.1337	0.2080	0.3471	0.091*	
C8	1.111 (2)	0.143 (3)	0.407 (4)	0.076 (6)*	
C9	1.036 (2)	0.125 (3)	0.447 (3)	0.076 (6)*	
H9	1.0524	0.0928	0.4716	0.091*	
C10	0.940 (3)	0.154 (3)	0.449 (3)	0.076 (6)*	
C11	0.854 (2)	0.135 (3)	0.487 (4)	0.076 (6)*	
C12	0.866 (3)	0.082 (3)	0.522 (3)	0.076 (6)*	
H12	0.9282	0.0589	0.5208	0.091*	
C13	0.786 (2)	0.066 (3)	0.556 (3)	0.076 (6)*	
H13	0.7939	0.0323	0.5801	0.091*	
C14	0.692 (2)	0.100 (3)	0.555 (3)	0.076 (6)*	
H14	0.6360	0.0897	0.5774	0.091*	
C15	0.683 (3)	0.149 (3)	0.518 (4)	0.076 (6)*	
H15	0.6206	0.1722	0.5166	0.091*	
C16	1.289 (3)	0.129 (3)	0.371 (3)	0.076 (6)*	
H16A	1.3137	0.1713	0.3801	0.091*	
H16B	1.2647	0.1291	0.3342	0.091*	
C17	1.375 (2)	0.081 (3)	0.379 (3)	0.076 (6)*	
C18	1.374 (2)	0.025 (3)	0.348 (3)	0.076 (6)*	
H18	1.3213	0.0190	0.3224	0.091*	
C19	1.450 (2)	−0.021 (4)	0.356 (4)	0.076 (6)*	
C20	1.533 (2)	−0.010 (3)	0.394 (4)	0.076 (6)*	
C21	1.534 (3)	0.045 (3)	0.424 (3)	0.076 (6)*	
H21	1.5854	0.0508	0.4500	0.091*	
C22	1.456 (3)	0.091 (3)	0.416 (3)	0.076 (6)*	
H22	1.4579	0.1287	0.4356	0.091*	
C23	1.374 (3)	−0.094 (3)	0.293 (3)	0.076 (6)*	
H23A	1.3070	−0.0869	0.3106	0.091*	
H23B	1.3750	−0.0699	0.2596	0.091*	
C24	1.386 (2)	−0.163 (3)	0.280 (3)	0.076 (6)*	
H24A	1.3360	−0.1759	0.2529	0.091*	
H24B	1.4564	−0.1714	0.2667	0.091*	
C25	1.425 (3)	−0.258 (3)	0.328 (3)	0.076 (6)*	
H25A	1.4982	−0.2514	0.3197	0.091*	
H25B	1.3957	−0.2859	0.2999	0.091*	
C26	1.413 (2)	−0.289 (3)	0.383 (3)	0.076 (6)*	
H26A	1.3382	−0.2938	0.3905	0.091*	
H26B	1.4423	−0.3316	0.3811	0.091*	
C27	1.575 (3)	−0.263 (3)	0.426 (3)	0.076 (6)*	
H27A	1.6059	−0.2454	0.3939	0.091*	
H27B	1.5957	−0.3071	0.4299	0.091*	
C28	1.604 (2)	−0.224 (3)	0.477 (4)	0.076 (6)*	
H28A	1.5510	−0.2319	0.5045	0.091*	
H28B	1.6703	−0.2398	0.4908	0.091*	
C29	1.700 (3)	−0.133 (4)	0.445 (4)	0.076 (6)*	
H29A	1.7112	−0.1511	0.4097	0.091*	
H29B	1.7629	−0.1398	0.4667	0.091*	
C30	1.675 (3)	−0.061 (3)	0.441 (4)	0.076 (6)*	
H30A	1.6398	−0.0464	0.4734	0.091*	
H30B	1.7384	−0.0367	0.4357	0.091*	
P1	0.9384 (18)	−0.019 (2)	0.343 (2)	0.106 (11)*	
F11	0.8318 (18)	0.013 (2)	0.360 (2)	0.183 (12)*	
F12	1.0448 (17)	−0.052 (2)	0.3259 (19)	0.183 (12)*	
F13	0.9814 (18)	−0.012 (2)	0.402 (2)	0.183 (12)*	
F14	0.984 (2)	0.046 (2)	0.327 (2)	0.183 (12)*	
F15	0.8942 (17)	−0.029 (2)	0.284 (2)	0.183 (12)*	
F16	0.8922 (18)	−0.086 (2)	0.359 (2)	0.183 (12)*	
O1W	0.2500	0.2500	0.287 (2)	0.120 (12)*	
H1W	0.2480	0.2200	0.2641	0.180*	
O2W	0.2037 (18)	0.154 (2)	0.206 (3)	0.106 (12)*	
H2W1	0.1545	0.1411	0.1858	0.159*	
H2W2	0.2413	0.1235	0.2171	0.159*	

### Geometric parameters (Å, °)

**Table:**

Cd—N1	2.42 (6)	C13—C14	1.39 (6)
Cd—N2	2.34 (3)	C14—C15	1.39 (10)
Cd—N3	2.39 (6)	C16—C17	1.51 (7)
Cd—N1^i^	2.42 (6)	C17—C18	1.41 (9)
Cd—N2^i^	2.34 (3)	C17—C22	1.40 (8)
Cd—N3^i^	2.39 (6)	C18—C19	1.38 (8)
P1—F15	1.58 (7)	C19—C20	1.43 (10)
P1—F16	1.56 (6)	C20—C21	1.38 (10)
P1—F12	1.57 (4)	C21—C22	1.39 (7)
P1—F13	1.57 (7)	C23—C24	1.50 (9)
P1—F11	1.58 (4)	C25—C26	1.51 (10)
P1—F14	1.56 (6)	C27—C28	1.55 (11)
O1—C16	1.50 (8)	C29—C30	1.56 (10)
O1—C8	1.32 (5)	C1—H1	0.93
O2—C23	1.42 (7)	C2—H2	0.93
O2—C19	1.37 (10)	C3—H3	0.93
O3—C24	1.43 (10)	C4—H4	0.93
O3—C25	1.45 (8)	C7—H7	0.93
O4—C26	1.40 (9)	C9—H9	0.93
O4—C27	1.47 (5)	C12—H12	0.93
O5—C29	1.40 (7)	C13—H13	0.93
O5—C28	1.34 (8)	C14—H14	0.93
O6—C20	1.34 (6)	C15—H15	0.93
O6—C30	1.44 (10)	C16—H16B	0.97
O1W—H1W	0.85	C16—H16A	0.97
O2W—H2W2	0.85	C18—H18	0.93
O2W—H2W1	0.85	C21—H21	0.93
N1—C5	1.36 (5)	C22—H22	0.93
N1—C1	1.34 (8)	C23—H23A	0.97
N2—C6	1.35 (8)	C23—H23B	0.97
N2—C10	1.37 (9)	C24—H24B	0.97
N3—C15	1.35 (8)	C24—H24A	0.97
N3—C11	1.35 (5)	C25—H25A	0.97
C1—C2	1.39 (9)	C25—H25B	0.97
C2—C3	1.40 (5)	C26—H26A	0.97
C3—C4	1.40 (9)	C26—H26B	0.97
C4—C5	1.40 (8)	C27—H27B	0.97
C5—C6	1.49 (9)	C27—H27A	0.97
C6—C7	1.42 (6)	C28—H28B	0.97
C7—C8	1.40 (10)	C28—H28A	0.97
C8—C9	1.43 (9)	C29—H29A	0.97
C9—C10	1.37 (5)	C29—H29B	0.97
C10—C11	1.50 (9)	C30—H30B	0.97
C11—C12	1.41 (10)	C30—H30A	0.97
C12—C13	1.38 (8)		
			
N1—Cd—N2	69.5 (17)	C19—C20—C21	119 (5)
N1—Cd—N3	139.8 (11)	C20—C21—C22	121 (6)
N1—Cd—N1^i^	96 (2)	C17—C22—C21	120 (6)
N1—Cd—N2^i^	107.5 (19)	O2—C23—C24	108 (4)
N1—Cd—N3^i^	97.7 (18)	O3—C24—C23	109 (6)
N2—Cd—N3	70.3 (17)	O3—C25—C26	109 (5)
N1^i^—Cd—N2	107.5 (19)	O4—C26—C25	117 (5)
N2—Cd—N2^i^	176 (3)	O4—C27—C28	101 (5)
N2—Cd—N3^i^	112.6 (18)	O5—C28—C27	116 (7)
N1^i^—Cd—N3	97.7 (18)	O5—C29—C30	106 (4)
N2^i^—Cd—N3	112.6 (18)	O6—C30—C29	104 (6)
N3—Cd—N3^i^	96 (2)	N1—C1—H1	119.02
N1^i^—Cd—N2^i^	69.5 (18)	C2—C1—H1	118.21
N1^i^—Cd—N3^i^	139.8 (11)	C3—C2—H2	120.64
N2^i^—Cd—N3^i^	70.3 (17)	C1—C2—H2	121.06
F13—P1—F15	179 (3)	C2—C3—H3	120.96
F13—P1—F16	89 (3)	C4—C3—H3	120.23
F14—P1—F15	90 (3)	C5—C4—H4	119.17
F14—P1—F16	180 (4)	C3—C4—H4	120.66
F15—P1—F16	90 (3)	C6—C7—H7	120.20
F11—P1—F12	179 (4)	C8—C7—H7	121.61
F11—P1—F13	90 (3)	C8—C9—H9	119.78
F11—P1—F14	90 (3)	C10—C9—H9	120.01
F11—P1—F15	90 (3)	C11—C12—H12	120.36
F11—P1—F16	90 (2)	C13—C12—H12	120.20
F12—P1—F13	90 (3)	C12—C13—H13	120.62
F12—P1—F14	90 (2)	C14—C13—H13	119.68
F12—P1—F15	90 (3)	C13—C14—H14	121.76
F12—P1—F16	91 (3)	C15—C14—H14	120.43
F13—P1—F14	91 (3)	N3—C15—H15	117.54
C8—O1—C16	120 (6)	C14—C15—H15	119.07
C19—O2—C23	118 (4)	O1—C16—H16A	109.92
C24—O3—C25	113 (5)	O1—C16—H16B	110.65
C26—O4—C27	113 (5)	C17—C16—H16A	109.67
C28—O5—C29	122 (5)	H16A—C16—H16B	108.72
C20—O6—C30	119 (7)	C17—C16—H16B	110.61
H1W—O1W—H1W^ii^	96	C17—C18—H18	119.46
H2W1—O2W—H2W2	111	C19—C18—H18	120.31
Cd—N1—C5	117 (4)	C22—C21—H21	120.53
Cd—N1—C1	123 (3)	C20—C21—H21	118.82
C1—N1—C5	120 (5)	C17—C22—H22	119.39
C6—N2—C10	120 (4)	C21—C22—H22	120.13
Cd—N2—C6	120 (4)	O2—C23—H23B	110.81
Cd—N2—C10	120 (3)	O2—C23—H23A	111.11
C11—N3—C15	119 (6)	H23A—C23—H23B	108.32
Cd—N3—C11	116 (5)	C24—C23—H23A	109.67
Cd—N3—C15	125 (3)	C24—C23—H23B	108.88
N1—C1—C2	123 (4)	O3—C24—H24A	109.30
C1—C2—C3	118 (6)	C23—C24—H24B	110.14
C2—C3—C4	119 (5)	O3—C24—H24B	109.01
C3—C4—C5	120 (4)	C23—C24—H24A	110.90
C4—C5—C6	123 (3)	H24A—C24—H24B	108.67
N1—C5—C4	120 (6)	O3—C25—H25B	109.47
N1—C5—C6	117 (5)	C26—C25—H25A	110.35
C5—C6—C7	122 (5)	C26—C25—H25B	111.70
N2—C6—C5	116 (3)	H25A—C25—H25B	107.65
N2—C6—C7	122 (6)	O3—C25—H25A	108.83
C6—C7—C8	118 (5)	O4—C26—H26A	108.81
O1—C8—C9	115 (7)	O4—C26—H26B	108.62
O1—C8—C7	126 (6)	C25—C26—H26B	107.92
C7—C8—C9	118 (4)	H26A—C26—H26B	106.66
C8—C9—C10	120 (6)	C25—C26—H26A	107.84
N2—C10—C9	121 (5)	O4—C27—H27A	111.94
C9—C10—C11	124 (6)	C28—C27—H27A	111.83
N2—C10—C11	115 (4)	C28—C27—H27B	111.22
N3—C11—C12	121 (5)	O4—C27—H27B	110.64
N3—C11—C10	118 (7)	H27A—C27—H27B	109.95
C10—C11—C12	121 (4)	C27—C28—H28A	108.37
C11—C12—C13	119 (4)	C27—C28—H28B	108.17
C12—C13—C14	120 (6)	H28A—C28—H28B	107.24
C13—C14—C15	118 (5)	O5—C28—H28B	108.40
N3—C15—C14	123 (4)	O5—C28—H28A	108.38
O1—C16—C17	107 (5)	C30—C29—H29A	111.02
C16—C17—C18	119 (5)	O5—C29—H29A	110.53
C16—C17—C22	122 (6)	O5—C29—H29B	110.18
C18—C17—C22	119 (5)	H29A—C29—H29B	108.73
C17—C18—C19	120 (6)	C30—C29—H29B	110.29
O2—C19—C18	125 (7)	O6—C30—H30B	111.22
C18—C19—C20	120 (7)	O6—C30—H30A	110.53
O2—C19—C20	115 (5)	H30A—C30—H30B	109.26
O6—C20—C19	114 (7)	C29—C30—H30A	110.60
O6—C20—C21	127 (6)	C29—C30—H30B	110.91

Symmetry codes: (i) −*x*+3/2, −*y*+1/2, *z*; (ii) −*x*+1/2, −*y*+1/2, *z*.

### Hydrogen-bond geometry (Å, °)

**Table:**

*D*—H···*A*	*D*—H	H···*A*	*D*···*A*	*D*—H···*A*
O1W—H1W···O2W	0.85	2.08	2.91 (6)	165
C3—H3···F15^iii^	0.93	2.44	3.21 (7)	140
C4—H4···O1W^iv^	0.93	2.23	3.17 (4)	178
C4—H4···O1W^i^	0.93	2.23	3.17 (4)	178
C7—H7···O1W^iv^	0.93	2.28	3.20 (7)	175
C7—H7···O1W^i^	0.93	2.28	3.20 (7)	175
C12—H12···F13^v^	0.93	2.44	3.10 (7)	128
C15—H15···O4^v^	0.93	2.51	3.23 (8)	135
C22—H22···O5^vi^	0.93	2.60	3.31 (10)	133
C23—H23B···F15^vii^	0.97	2.36	3.23 (8)	149
C30—H30B···F11^iv^	0.97	2.46	3.23 (8)	137

Symmetry codes: (iii) −*x*+2, *y*+1/2, −*z*+1/2; (iv) *x*+1, *y*, *z*; (i) −*x*+3/2, −*y*+1/2, *z*; (v) −*x*+2, −*y*, −*z*+1; (vi) −*x*+3, −*y*, −*z*+1; (vii) *x*+1/2, −*y*, −*z*+1/2.
